# Supplementary material for: What Drives Chorismate Mutase to Top Performance? Insights from a Combined In Silico and In Vitro Study
Source: Biochemistry. 2023 Jan 27;62(3):782–96. doi: 10.1021/acs.biochem.2c00635 (PMC9910054; doi:10.1021/acs.biochem.2c00635)
Supplement: Supplementary file 1 — bi2c00635_si_001.pdf [file bi2c00635_si_001.pdf]

SUPPORTING INFORMATION

What drives chorismate mutase to top performance? Insights from a combined *in silico* and *in vitro* study

Helen V. Thorbjørnsrud[1,2#], Luca Bressan[3#], Tamjidmaa Khatanbaatar[1,2#], Manuel Carrer[1,2], Kathrin Würth-Roderer[3], Gabriele Cordara[1,2], Peter Kast[3*], Michele Cascella[1,2*], Ute Krengel[1,2*]

[1] Department of Chemistry, University of Oslo, NO-0315 Oslo, Norway

[2] Hylleraas Centre for Quantum Molecular Sciences, University of Oslo, NO-0315 Oslo, Norway

[3] Laboratory of Organic Chemistry, ETH Zurich, CH-8093 Zurich, Switzerland

[#]H.V.T., L.B. and T.K. contributed equally to this work.

*corresponding authors: kast@org.chem.ethz.ch, michele.cascella@kjemi.uio.no, ute.krengel@kjemi.uio.no

LIST OF MATERIAL INCLUDED:

Figures S1-S5 (S1, Crystal structures and electron density; S2, Crystal contacts; S3 RMSF plot; S4-5, MD snapshots)

Table S1 (Data collection and refinement statistics)

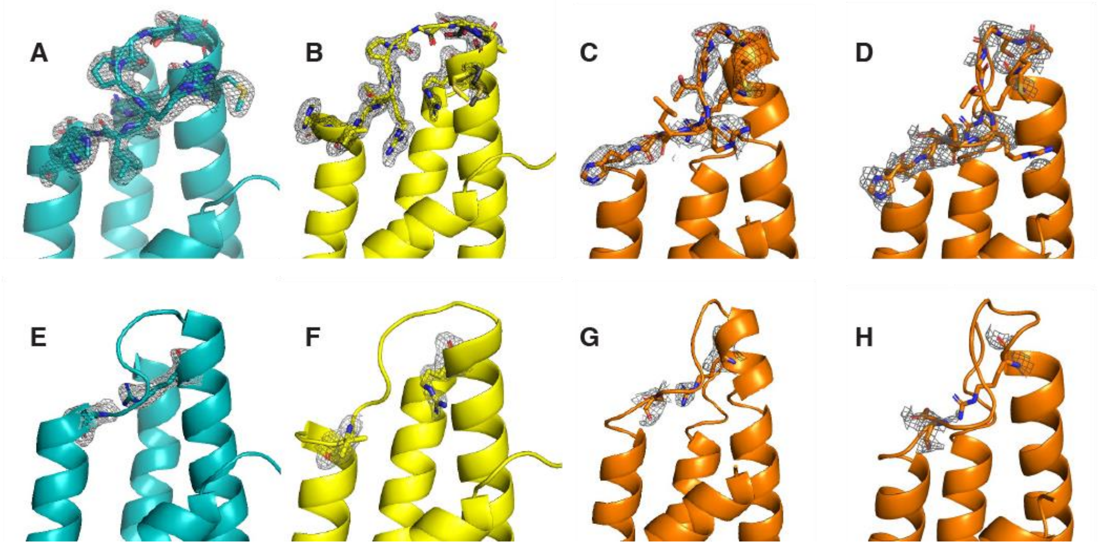

Figure S1. Crystal structures and electron density maps shown for catalytically important regions. Top row, active site H1-H2 loop residues (^{47}MASGGPRLVHS^{57}). Bottom row, residue 55 and the catalytically crucial Arg46. **(A)** and **(E)**, top-evolved MtCM^{V} (PDB ID: 5MPV;[1] cyan); **(B)** and **(F)**, MtCM^{T52P} (PDB ID: 6YGT, this work; yellow); **(C)** and **(G)**, MtCM^{V55D}, protomer A (this work; orange); **(D)** and **(H)**, MtCM^{V55D}, protomer B (this work; orange). The MtCM^{V55D} structure is of low quality, precluding final refinement; therefore, the coordinates were not deposited in the PDB. All electron density maps are σ_A-weighted 2m*F*o-D*F*c maps, depicted at σ =1.0.

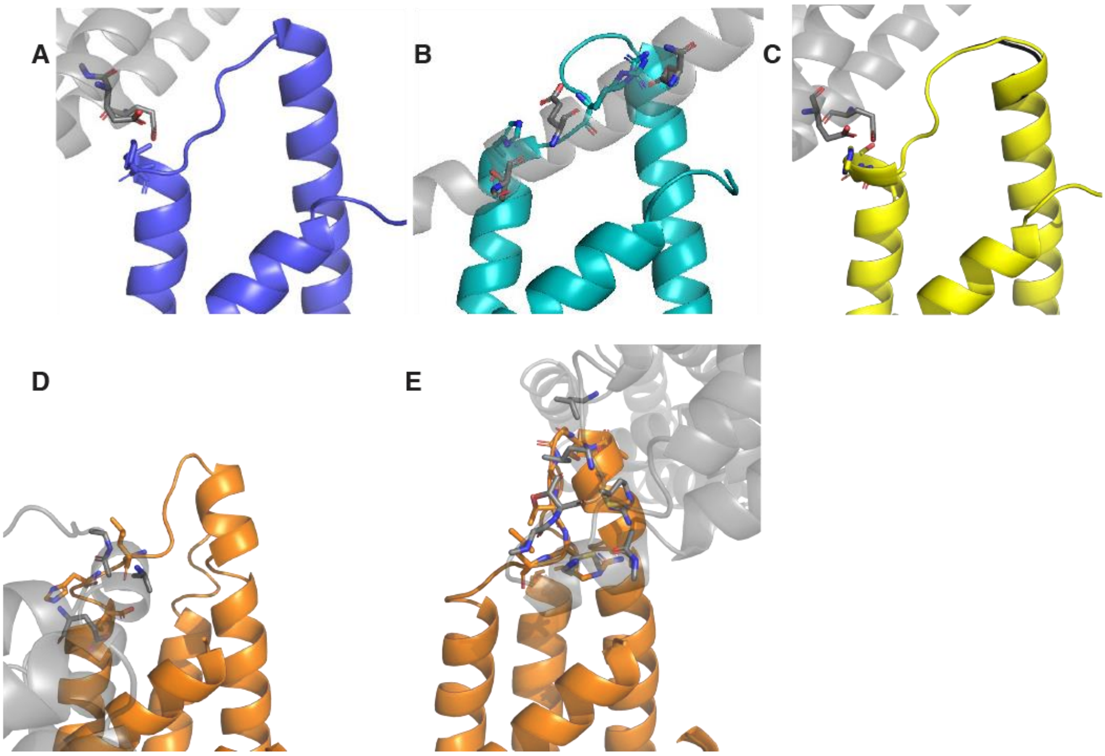

Figure S2. Crystal contacts of the H1-H2 loop with the closest neighboring molecule (shown in grey). (A) Wild-type MtCM (PDB ID: 2VKL;[2] purple), **(B)** Top-evolved MtCM^{V} (PDB ID: 5MPV;[1] cyan), **(C)** MtCM^{T52P} (PDB ID: 6YGT, this work; yellow), **(D)** MtCM^{V55D}, protomer A (this work; orange), **(E)** MtCM^{V55D}, protomer B (this work; orange). The structures in panels A and C have the same crystal form.

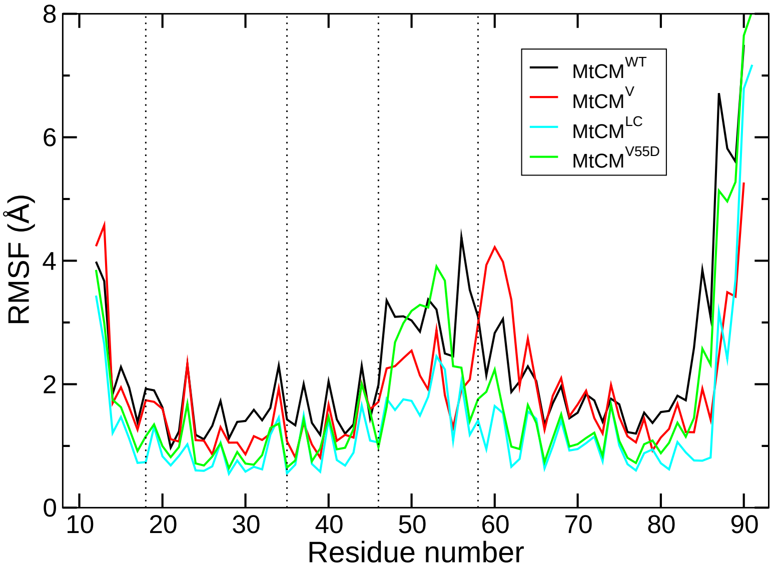

Figure S3. Root mean square fluctuations of MtCM during MD simulations. The graph reports the mean RMSF values averaged per residue for MtCM^{WT} and the super-active variant MtCM^{V} as well as for the ligand complex MtCM^{LC} and the MtCM^{V55D} single variant. The vertical dotted lines indicate the positions of the four key arginine residues listed in Table 1.

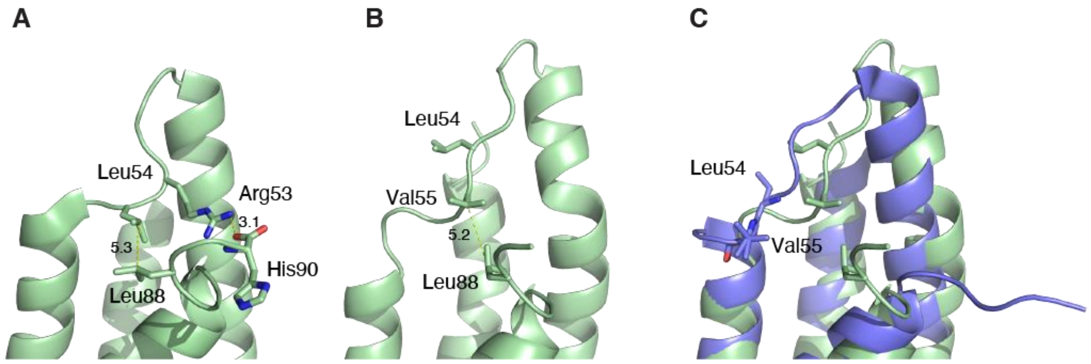

Figure S4. Interactions in MtCM between its C-terminus and H1-H2 loop. (**A**) Cartoon illustration of MtCM^{DS} (MtCM from the MtCM-MtDS crystal structure; PDB ID: 2W19[2]). MtCM^{DS} is colored green and residues involved in prominent interactions are shown as sticks. Two interactions are highlighted: hydrophobic contacts between Leu54 of the H1-H2 loop and Leu88 close to the C-terminus of MtCM (distance measured between C_γ of Leu side chains), and a salt bridge between Arg53 of the H1-H2 loop and the C-terminal carboxylate (His90). (**B**) Cartoon illustration of van der Waals interactions observed for chain A of MtCM^{DS} at time step 250 (25 ns simulation). Note the distinct conformation of the H1-H2 loop, with Val55 temporarily taking over the role of Leu54. However, this conformational change is catalytically unfavorable, as it interferes with substrate binding to the Val55 main chain amide group, as shown in Fig. 1F. (**C**) Superimposition of MtCM^{DS} (simulated structure from **B**, green) with MtCM crystal structure (purple, PDB ID: 2VKL[2]). The corresponding residues Val55 and Leu54 in the two structures, shown as sticks, occupy shifted positions, but in similar (catalytically unfavorable) orientations in the respective H1-H2 loops.

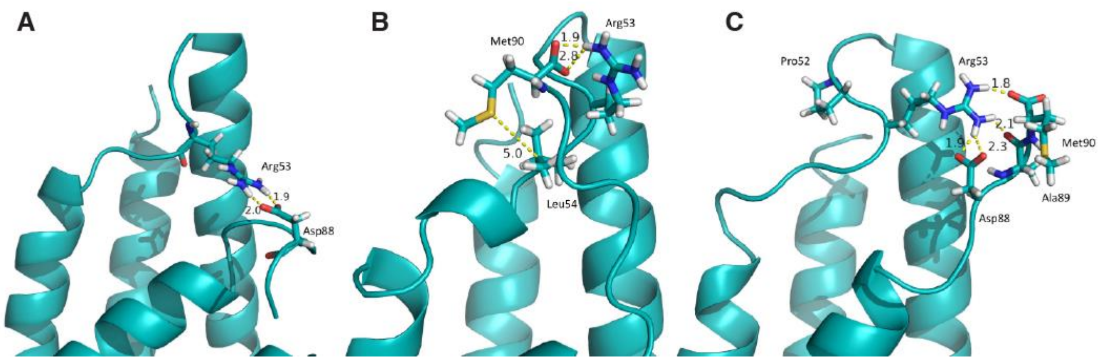

Figure S5. MD snapshots of interactions between C-terminus and H1-H2 loop of MtCM^{V}. The C-terminus is very flexible, but recurring interactions are made by carboxylic acid groups from the C-terminal sequence (C-terminal carboxylate or Asp88 side chain) to Arg53 of the H1-H2 loop. Representative frames are shown in cartoon representation, with relevant residues as sticks. (**A**) Chain A at 15.1 ns, (**B**) Chain B at 17.8 ns, (**C**) Chain A at 4.1 ns (intermediate conformation).

Table S1 – Data collection and refinement statistics

	$MtCM^{T52P}$	$MtCM^{V55D}$
Data collection		
Beamline	ESRF ID3-A30/MASSIF-3	ESRF ID29
Wavelength (Å)	0.9677	0.9753
Space group	$P4_32_12$	$P2\ 2_1\ 2_1$
Cell parameters - *a*, *b*, *c* (Å)	59.6, 59.6, 46.6	32.2, 59.7, 72.1
Protein chains in a.s.u.	1	2
Matthew's coefficient ($Å^3$/Da)	2.0	1.7
Resolution (Å)[a]	36.7-1.64 (1.72-1.64)	45.94-2.06 (2.25-2.06)
$CC_{1/2}$ (%)[a, b]	99.5 (45.1)	99.9 (47.6)
Mean I/σ(I)[a]	15.7 (1.1)	14.6 (1.1)
Completeness (%)[a]	88.1 (33.4)	82.4 (22.3)
Number of unique reflections [a]	9570 (479)	7445 (438)
Multiplicity[a]	8.7 (9.8)	6.0 (6.1)
Wilson B-factor ($Å^2$)	22.2	57.8
Refinement[d]		
Resolution range (Å)	29.8-1.64	
R_{work}/R_{free} (%)[c]	24.0/26.5	
Average *B*-factor ($Å^2$)	32.6	
Number of atoms		
Protein	674	
Water	18	
r.m.s.d. from ideal geometry		
Bond lengths (Å)	0.01	
Bond angles (deg.)	1.2	
Ramachandran plot[e]		
Favored (%)	93.1	
Allowed (%)	5.2	
Outliers (%)	0.0	
PDB code	6YGT	

[a] Values in parentheses refer to highest resolution shell. The data completeness falls below the 95% threshold beyond ~1.85 Å and ~2.40 Å resolution for $MtCM^{T52P}$ and $MtCM^{V55D}$, respectively, which can be considered the effective resolution of the data sets.

[b] Reflections up to the highest resolution limit were included assessing the data using the $CC_{1/2}$ parameter, as suggested by Diederichs and Karplus.[3,4]

[c] R_{free} was calculated from 5% of randomly selected reflections for each data set.

[d] Refinement of $MtCM^{V55D}$ stalled at R_{work}/R_{free} values of 27.6/34.9%. The structure was therefore not included in this Table or submitted to the PDB.

[e] Calculated with *SFCHECK*.[5] Ser49 (and Gly51) have torsion angles bordering to outlier regions in the Ramachandran plot, and were identified as outliers by the PDB.

Supporting References

(1) Fahrig-Kamarauskaitė, J.; Würth-Roderer, K.; Thorbjørnsrud, H. V.; Mailand, S.; Krengel, U.; Kast, P. Evolving the naturally compromised chorismate mutase from *Mycobacterium tuberculosis* to top performance. *J. Biol. Chem.* **2020**, *295*, 17514-17534.

(2) Sasso, S.; Ökvist, M.; Roderer, K.; Gamper, M.; Codoni, G.; Krengel, U.; Kast, P. Structure and function of a complex between chorismate mutase and DAHP synthase: Efficiency boost for the junior partner. *EMBO J.* **2009**, *28*, 2128-2142.

(3) Diederichs, K.; Karplus, P. A. Better models by discarding data? *Acta Crystallogr. D Biol. Crystallogr.* **2013**, *69*, 1215-1222.

(4) Karplus, P. A.; Diederichs, K. Assessing and maximizing data quality in macromolecular crystallography. *Curr. Opin. Struct. Biol.* **2015**, *34*, 60-68.

(5) Vaguine, A. A.; Richelle, J.; Wodak, S. J. *SFCHECK*: A unified set of procedures for evaluating the quality of macromolecular structure-factor data and their agreement with the atomic model. *Acta Crystallogr. D Biol. Crystallogr.* **1999**, *55*, 191-205.
